# Decremental evoked potentials’ sites characterized by low voltage and consistent electrograms amplitude in patients with post-infarction ventricular tachycardia

**DOI:** 10.1007/s10840-026-02270-5

**Published:** 2026-03-18

**Authors:** Ahmed S. Yassin, Alwin B. P. Noordman, Alexander H. Maass, Michiel Rienstra, Yuri Blaauw

**Affiliations:** Department of Cardiology, University of Groningen, University Medical Center Groningen, Groningen, The Netherlands

**Keywords:** Ventricular tachycardia, Voltage mapping, Electroanatomical mapping, Substrate mapping, Decremental evoked potentials

## Abstract

**Background:**

Functional substrate mapping during infarct-related ventricular tachycardia ablation is a crucial step to identify critical isthmus locations. The use of basic voltage map metrics to predict the location of these critical sites might alleviate the challenges of performing full left ventricle functional mapping and might facilitate success and efficiency of infarct-related VT ablation.

**Objective:**

This study aims to characterize regional voltage metrics at sites showing Decremental Evoked Potentials (DEEP) during functional mapping and also aims to detect whether these voltage metrics can potentially predict DEEP sites.

**Methodology:**

This is a retrospective study wherein 14 patients with infarct-related VT underwent endocardial ablation in our institution between January 2021 and March 2023. High-density omnipolar voltage and functional substrate mapping maps were exported and merged with a cardiac tomography (CT)-derived left ventricular (LV) model. Subsequently these models were segmented into 17 segments (per patient) for further analysis. For each segment values of voltage median, voltage interquartile range (IQR), and presence of DEEP response during functional mapping were acquired. The difference in these voltage metrics between DEEP + and DEEP- segments was analyzed. Furthermore, the ability of these metrics to predict DEEP status was explored.

**Results:**

A total of 233 LV segments were included. 168 segments were labelled as DEEP negative (DEEP-). The DEEP+ segments exhibited lower voltage than DEEP – segments (respectively 0.94 mV versus 1.95 mV, *p* < 0.001) and lower voltage interquartile range (IQR) (respectively 1.01 mV versus 2.66 mV, *p* < 0.001). Furthermore, each 1-mV increase in any segment voltage median was associated with a 53% reduction in the odds of DEEP+ (OR = 0.47, 95% CI [0.33, 0.66], *p* < 0.001), and a 1-mV increase in voltage IQR was associated with a 45% decrease (OR = 0.55, 95% CI [0.42, 0.72], *p* < 0.001). The Receiver Operating Characteristic (ROC) analysis revealed that the AUC for voltage median was 0.70 and for voltage IQR was 0.71. The optimal thresholds for predicting DEEP+ sites were voltage median < 1.53 mV and voltage IQR < 2.55 mV.

**Conclusions:**

Left ventricular regions harboring critical electrograms displaying a DEEP+ response tend to have lower regional voltage median and lower voltage IQR.

## Introduction

Infarct-related ventricular tachycardia (VT) is most commonly reentrant in mechanism [1]. This reentrant circuit utilizes a critical arrhythmogenic substrate that features: low voltage electrograms, slow conduction, functional or fixed block, abnormal late potentials, and fractionated electrograms with prolonged duration [2]. VT ablation is an established treatment in patients with recurrent VTs [3]. The ablation success requires precise and accurate determination of the critical VT substrate. Hence, substrate mapping is a fundamental step to delineate and understand this critical arrythmia substrate [4]. The recently described functional substrate mapping method, Decremental Evoked Potential mapping (DEEP), has gained wide validation to serve the above purpose [5]. DEEP mapping is performed by delivering an extra stimulus at areas of interest to observe whether a decremental response in existing or hidden late potentials occurs. Performing this mapping method remains challenging due to a chance of non-hemodynamically tolerable VT induction, interpretation challenges, and manual annotation [6]. More static and basic mapping methods like voltage mapping are routinely acquired during VT ablation procedures [7]. The ability to utilize the voltage map to predict the potential regional presence of functional substrate (i.e. DEEP) would facilitate functional mapping and refine substrate characterization by targeting and focusing on the left ventricular (LV) regions with distinctive voltage characteristics that may harbour these critical circuits. This study aims to analyse the regional LV voltage characteristics by evaluating the association between DEEP presence and two key voltage metrics: the regional voltage median, representing the overall voltage amplitude, and the voltage interquartile range (IQR), a measure of regional voltage heterogeneity.

## Methodology

### Patient population

This retrospective study enrolled consecutive patients who presented to our institution for elective endocardial ablation of infarct-related VT between January 2021 and March 2023. The inclusion criteria included all the following: adult patients, underwent preoperative cardiac CT scan, and had intraoperative high-density electroanatomical mapping (EAM) with S1 and S2 extra stimulus. Patients with unavailable/non-stored EAM or poor-quality CT were excluded. A total of 14 patients were successfully enrolled in the final analysis. The local ethical committee approved the study, and written informed consent was acquired from all alive participants.

### Endocardial VT ablation and high-density LV mapping

The ablation procedure was performed under deep sedation. LV access was achieved via a transseptal route or retro-aortic approach. Diagnostic catheters were placed in the coronary sinus and right ventricle as needed. Ventricular tachycardia (VT) induction was attempted using programmed electrical stimulation. Activation mapping, entrainment mapping, and subsequent pace mapping was performed in successfully induced VT once feasible. Endocardial LV electroanatomical mapping (EAM) was obtained using HD Grid mapping catheter (EnSite™ X system). Detailed mapping of the LV was performed during fixed drive train RV apex pacing (500-600ms). Additional RV apex S2 extrastimulus mapping (50ms above the ventricular effective refractory period (VERP)) was simultaneously performed in all cases. Finally, radiofrequency ablation was applied targeting potential critical VT isthmuses that were identified by combining information obtained by VT activation mapping, activation map of S1, DEEP locations and pace mapping data. The procedural endpoint was defined as non-inducibility of the clinical VT.

### Postprocedural analysis

**Fig. 1:**
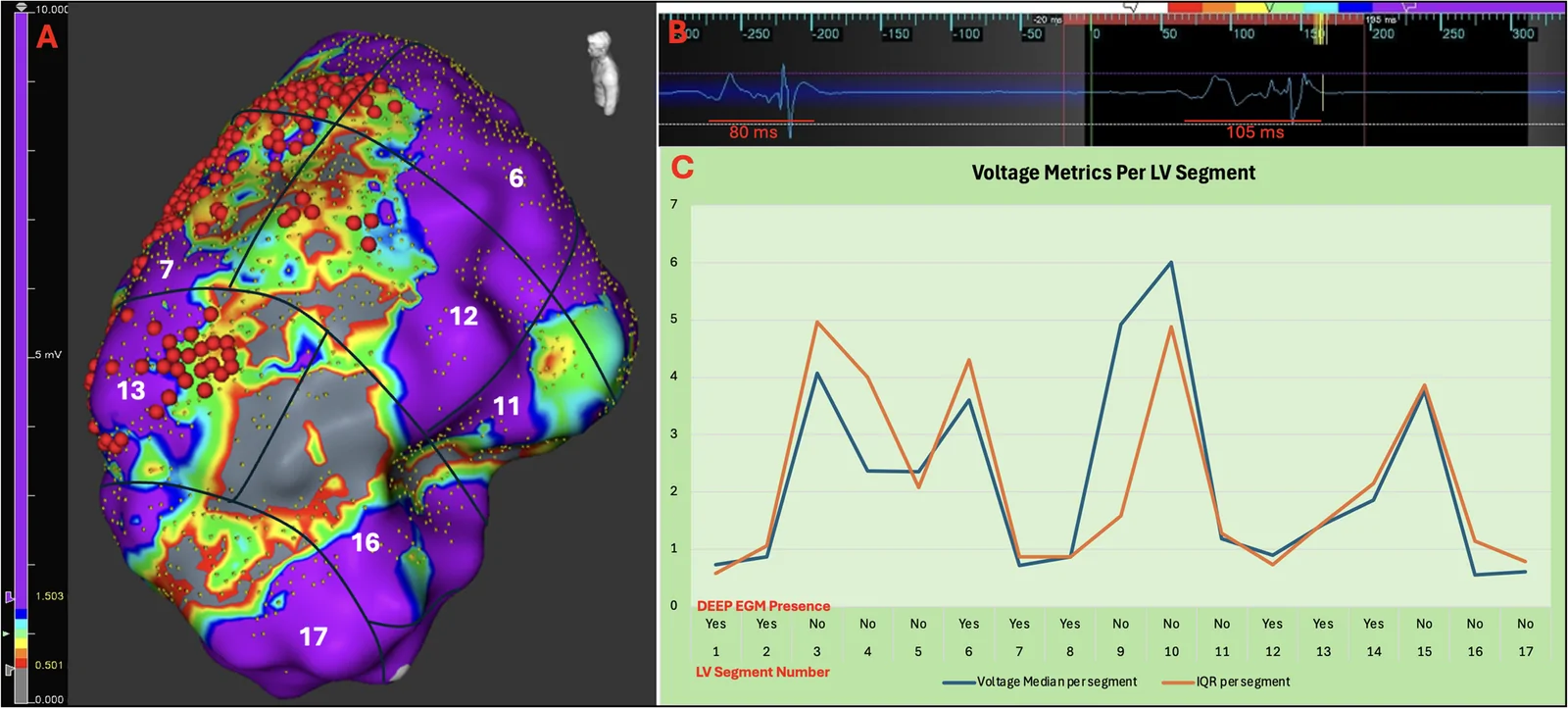
Display of the processing of an example case. **A** shows an export of LV S1 voltage map to ADAS 3D software, with red dots to reflect the location of positive DEEP signals. LV segmentation in this figure was created manually for the purpose of illustration only. **B** Electrogram showing DEEP+ response. **C** graphic display of regional voltage metrics per LV segment (1–17) and the corresponding LV segment DEEP status (Yes/No). DEEP, decremental evoked potentials; EGM, electrogram; IQR, interquartile range; LV, left ventricle

For this study, a retrospective analysis of the cases was performed. The EAM was reviewed and adjusted to ensure proper annotation and remove artifacts. Two models were created: (1) an S1 voltage map (Omnipolar), and (2) S2 DEEP map. For the latter, electrograms (EGMs) that displayed DEEP response during S2 mapping were labelled and displayed in the LV EAM Omnipolar voltage map model. If the local LP electrogram delayed by > 20 ms, the LP was labelled as DEEP+ [5, 8]. The resultant EAM models were exported to a dedicated LV segmentation software (ADAS 3D), where the models were registered with LV computed tomography (CT) model to facilitate the subsequent LV segmentation of each LV into 17 segments. 233 LV segments were used in the final analysis (5 segments were excluded due to paucity of mapping points precluding voltage metrics calculations). For the S1 Omnipolar voltage map two metrics were defined to describe the voltage status of each LV segment: the voltage median which provides a central tendency of electrogram amplitude to classify tissue as *healthy voltage zone* (> 1.5 mV), *border zone* (reduced voltage zone; ≥ 0.5–1.5 mV) or *severely reduced voltage zone* (< 0.5 mV), and the voltage IQR (interquartile range) of each LV segment which quantifies the dispersion of recorded electrogram amplitudes, reflecting the local voltage amplitude variation. In addition, LV segments were defined as DEEP positive (DEEP+) if more than 10 EGMs showed decremental late potential delay response (Fig. 1).

### Statistical analysis

Shapiro Wilks test was used to test for normality. All normally distributed continuous variables were reported as mean (± standard deviation (SD)), while non-normally distributed continuous variables were presented as median (IQR). Due to departure from normality of each segment LV voltage values, voltage median and IQR were used to represent the voltage blueprint of each segment. Categorical variables were reported as numbers and percentages of the total. Non-parametric Wilcoxon-Mann-Whitney test was utilized to assess the differences in both median voltage and IQR between DEEP-positive and DEEP-negative segments. Furthermore, linear mixed-effects models were performed to validate the comparison, while accounting for the non-independence of LV segments sampled from each individual patient. A mixed-effects logistic regression was performed to obtain odds ratios. Finally, a Receiver Operating Characteristic (ROC) analysis was performed to estimate the optimal thresholds for voltage median and voltage IQR per LV segment to determine segment DEEP status. The optimal thresholds were estimated using Youden’s J statistic to handle class imbalance. Youden’s J equally weights sensitivity and specificity, doesn’t assume equal class distributions, and finds the threshold that gives the best balance between true positive and true negative rates. The statistical analysis was performed using R software (v4.3.1). A p value of 0.05 was set to determine statistical significance.

## Results

### Cohort baseline

The mean age was 69 ± 7 years. Mean left ventricular ejection fraction (LVEF) was 35% (± 10). All patients had a history of previous myocardial infarction, with 3 (21.4%) having anterior wall infarct and 11 (78.6%) having inferior wall infarct. All patients had an implantable cardioverter defibrillator (Table 1).

**Table 1:** Baseline characteristics and relevant procedure metrics

Age (years)	69 (± 7)
Female	1 (7.1%)
BMI (kg/m^2^)	26.8 (23.6 – 30.0)
ICD	14 (100.0%)
CAD	14 (100.0%)
Hypertension	9 (64.3%)
Atrial Fibrillation	4 (28.6%)
Diabetes Mellitus	6 (42.9%)
Severely Reduced Voltage Zones’ Location:	
Anterior Wall	3 (21.4%)
Inferior Wall	11 (78.6%)
Prior PCI	9 (64.3%)
Prior CABG	4 (28.6%)
Beta-blocker	14 (100.0%)
Antiarrhythmic medication (class 3)*	11 (78.6%)
LVEF (%)	35 (± 10)
eGFR (mL/min/1.73m^2^)	53 (43 – 81)
Preprocedural CT	14 (100.0%)
Endocardial ablation	14 (100.0%)
Procedure time (minutes)	217 (198 – 254)
VT inducible	
Inducible	12 (85.7%)
Not Inducible	1 (7.1%)
No induction performed	1 (7.1%)
Clinical VT	
Inducible	8 (57.1%)
Not Inducible	4 (28.6%)
No induction performed	1 (7.1%)
Hemodynamically tolerated VT	7 (50.0%)
VT map	7 (50.0%)
Omnipolar Voltage map	14 (100.0%)
Functional substrate map	14 (100.0%)
Pace map	13 (92.9%)
No inducible VT at end of procedure	6 (42.9%)
Inducible clinical VT at end of procedure	1 (7.1%)

*Antiarrhythmic medication (class 3) includes sotalol and amiodarone. BMI, body mass index; CABG, coronary artery bypass graft; CAD, coronary artery disease; CT, computed tomography; eGFR, estimated glomerular filtration rate; ICD, implantable cardioverter defibrillator; LV, left ventricle; LVEF, left ventricular ejection fraction; PCI, percutaneous coronary intervention; VT; ventricular tachycardia

### Procedure and high-density mapping

Omnipolar voltage mapping and DEEP mapping were performed in all patients. The mean number of S1 mapping points per LV was 2756 points/map (± 941). The mean number of S2 mapping points was 1624 points/map (± 908). Of the 233 LV segments, 168 segments were labelled as DEEP negative (DEEP-). The mean recorded voltage was 1.02 mV (± 0.45) at the DEEP+ locations, and 3.34 mV (± 1.44) at the DEEP- locations.

### Direct comparison of voltage metrics between DEEP + and DEEP- segments

**Fig. 2:**
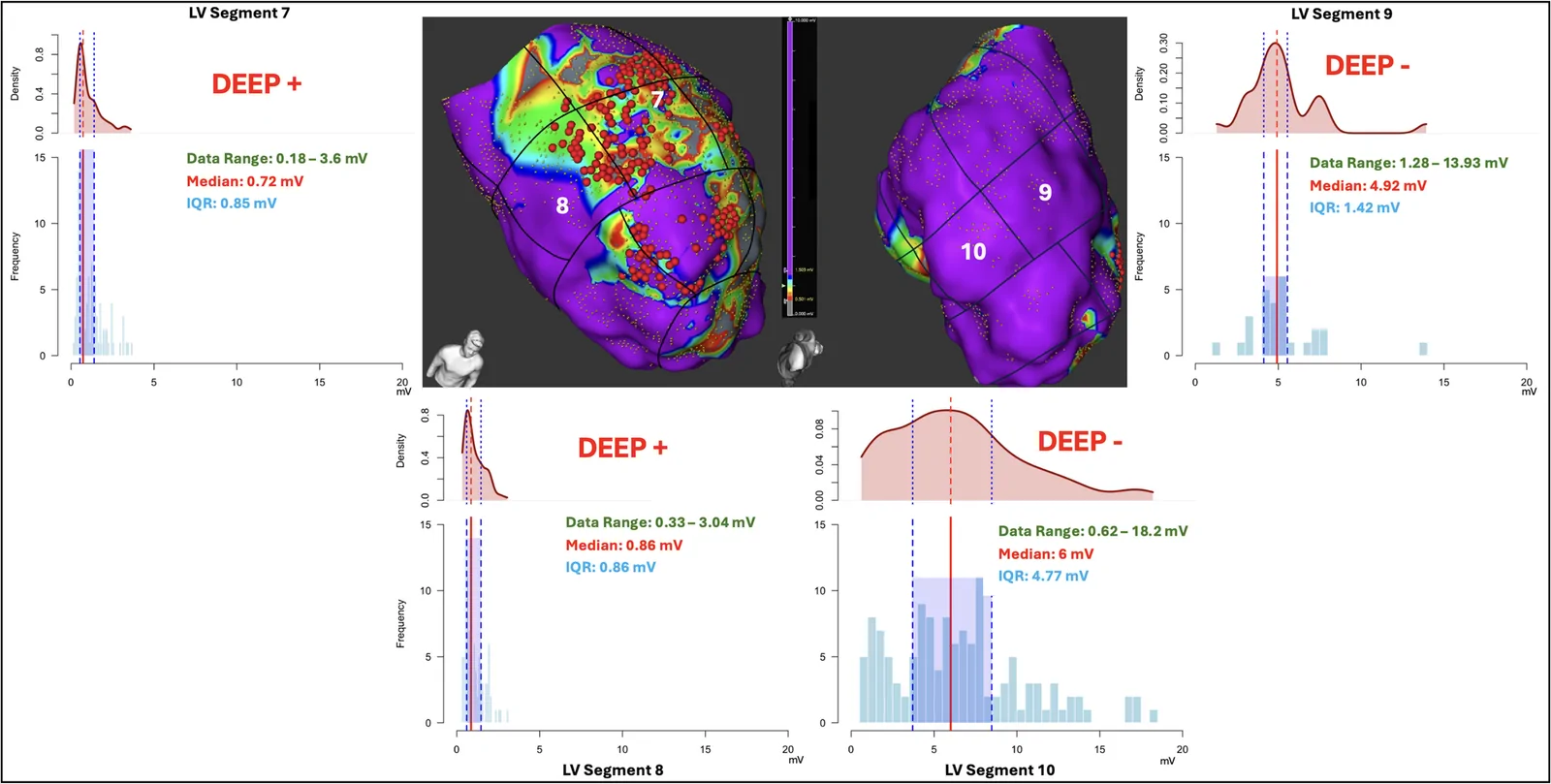
Illustration of the segmental voltage parameters with DEEP status. Center of the figure; represents a segmented left ventricle model with a projected voltage map with red dots indicating the location of DEEP. Peripheral graphs: represent voltage measurement distribution among four different left ventricular segments (two segments are DEEP +, and two segments are DEEP -). The top graph for each segment displays a probability density plot, which provides a representation of the voltage distribution per segment. The height at any point indicates the probability (Y-axis) of observed voltage values (x-axis) in that range. The bottom of the panels shows a histogram graph, which displays the actual quantity (y-axis) of measurements within each voltage range (x- axis). The red vertical line indicates the voltage median, while the blue dashed lines point out the first and third quartiles. DEEP, decremental evoked potentials; EGM, electrogram; IQR, interquartile range; LV, left ventricle. LV segmentation in this figure was created manually for the purpose of illustration only

The DEEP+ segments exhibited substantially lower voltage median (0.94 mV (0.65–1.75) versus 1.95 mV (1.01–3.38), *p* < 0.001) and lower voltage IQR per LV segment (1.01 mV (0.68–2.12) versus 2.66 mV (1.21–4.01), *p* < 0.001) compared to DEEP- segments. DEEP + segments were observed in 20 (15.87%) of segments with healthy voltage range (voltage median > 1.5 mV), in 35 (44.87%) of border zone (reduced voltage zone) segments (≥ 0.5–1.5 mV), and in 10 (34.48%) of severely reduced voltage segments (< 0.5 mV). The mixed-effects model confirmed the persistence of the significant difference after accounting for clustered data effect (i.e. every 17 segments originate from the same patient), where DEEP+ segments were found to have a voltage median that is 1.24 mV lower than DEEP- segments (β = -1.24 mV, 95% CI [-1.69- -0.79], *p* < 0.001), and IQR that is 1.23 mV lower than DEEP- segments (β = -1.23 mV, 95% CI [-1.70 - -0.75], *p* < 0.001) (Fig. 2).

### Voltage metrics as predictors of DEEP+ segments

**Fig. 3:**
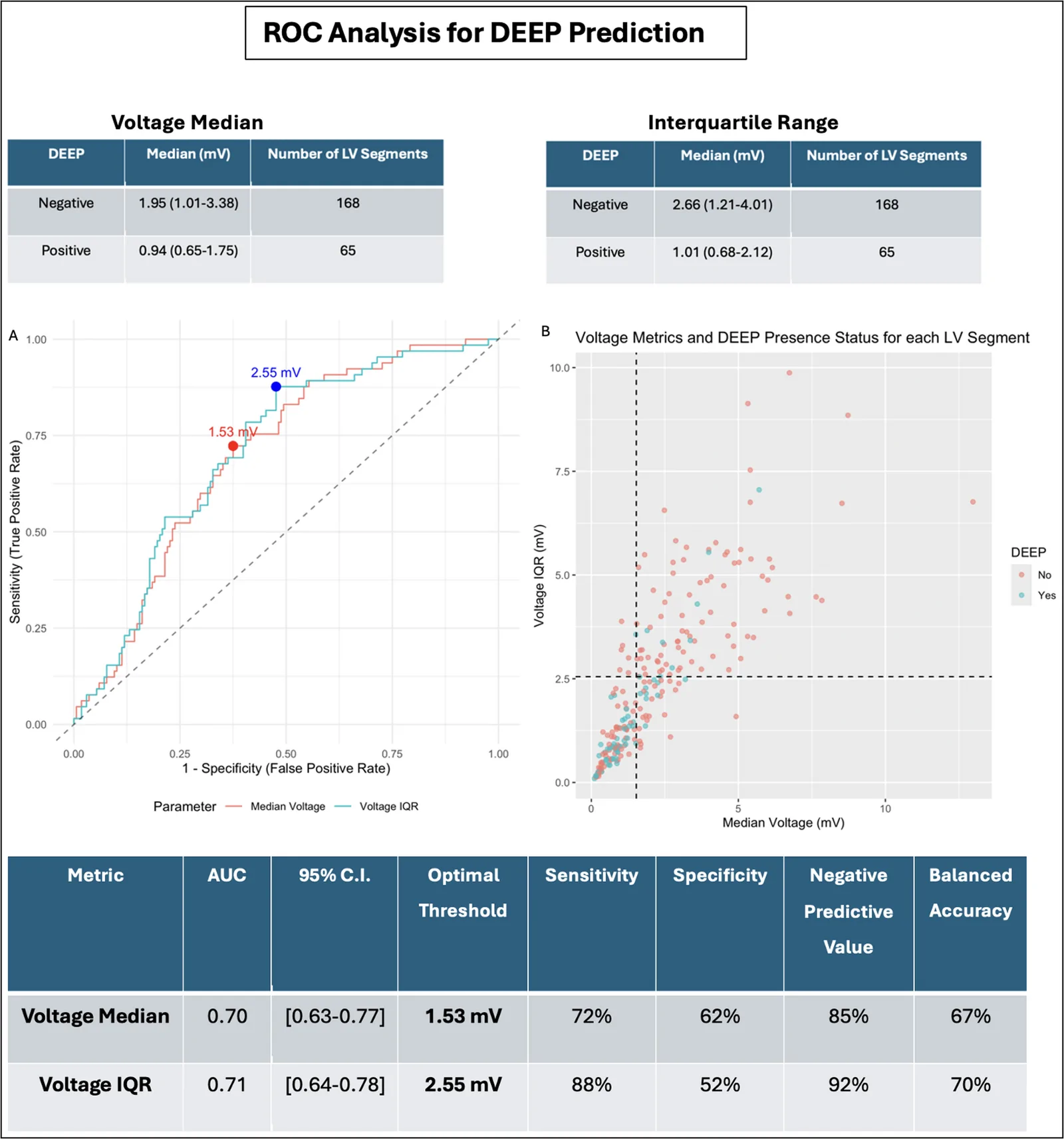
Results of the Receiver Operating Characteristic analyses. A; shows ROC for both voltage parameters. B; plot representing the entire sample of LV segments displaying voltage metrics per DEEP status. Tables are titled. CI, confidence interval; DEEP, decremental evoked potentials; EGM, electrogram; IQR, interquartile range; LV, left ventricle; ROC, receiver operating characteristic

Mixed-effects multilevel logistic regression confirmed that both lower voltage median and lower IQR were predictors of increased probability of DEEP+ identification. Each 1-mV increase in a LV segment’s voltage median was associated with a 53% reduction in odds of being DEEP+ segment (OR = 0.47, 95% CI [0.33, 0.66], *p* < 0.001), and a 1-mV increase in voltage IQR was associated with a 45% reduction (OR = 0.55, 95% CI [0.42, 0.72], *p* < 0.001). The ROC analysis revealed that the performance of these defined voltage metrics to discriminate between DEEP + and DEEP- segments was reasonable. The area under the curve (AUC) for voltage median was 0.70 and for IQR was 0.71. The optimal thresholds for predicting DEEP sites were voltage median < 1.53 mV and IQR < 2.55 mV (Fig. 3).

## Discussion

The analyses demonstrated a significant association between DEEP presence in a given LV segment with voltage median and voltage IQR. These findings point out that DEEP+ sites are characterized by significantly reduced voltages and less variable amplitude (low IQR) which is consistent with an electrically distinctive and inert substrate.

The IQR has been described as a measure of statistical dispersion [9]. It measures the spread of the middle 50% of a dataset (i.e. voltage values per LV segment in our analysis) [10]. Tissue in severely-reduced voltage zones and border zone has low voltage values (respectively 0.00- < 0.50 and ≥ 0.50–1.50 mV [11]) and a low range (i.e. voltage range 0.00–1.50 mV), which would result in a low IQR. Leveraging this basic fact at the segment level would be more appropriate than using the voltage median alone because, despite the outliers being accounted for during the calculation of the median, yet the representation of the data distribution remains missing, suggesting that the utilization of both variables would be more comprehensive. On the other hand, the healthy myocardium exhibits higher voltage values (> 1.50 mV) and inherent higher voltage range (i.e. voltage range of > 1.50 – ~20 mV) [12]. This variability and higher range in absolute values would be expected to result in a higher voltage IQR within a defined LV segment.

DEEP mapping, despite being highly specific, can be time-consuming and requires operative experience for immediate interpretation. Furthermore, DEEP dedicated pacing protocols and maneuvers are subjected to challenging factors, like proper pacing train capture, pacing site in relation to the severely-reduced voltage zones’ location, subsequent activation direction, and any in-between conduction barriers [13]. Voltage mapping during fixed train pacing is routine practice, easy to acquire and interpret, and less subject to challenges associated with DEEP pacing. It provides a static and anatomical representation of the severely reduced voltage zones (< 0.5 mV) and border zones (reduced voltage zones; ≥ 0.5–1.5 mV). The convergence of persistent and low amplitude voltage metrics (i.e. low voltage and lower voltage IQR) is particularly indicative of severely reduced voltage zones with limited viable myocardium. This electrically inert environment creates the required circumstances for slow conduction and wavefront block, which are the fundamental mechanisms enabling the formation of functional substrate characteristics like decremental evoked potentials. Our findings align with several studies. A recent study to evaluate the additional value of vector field heterogeneity showed that critical isthmuses harbour lower Omnipolar voltage in infarct-related VT. Voltage measurements showed a clear gradient: the critical isthmus site (0.25 ± 0.42 mV) and adjacent low-voltage bystander areas (0.26 ± 0.36 mV) exhibited similarly low voltages, which were substantially lower than the 6.48 ± 4.35 mV recorded in normal bystander myocardium [14]. Another study used high-density mapping of VT circuits related to scars to examine and characterize the topography of VT isthmuses as well as local conduction velocity and voltages. Thirty-six complete VT circuits in 31 patients were identified. The median of the amplitude of near-field diastolic electrograms in the mid-section of the isthmus during sinus rhythm or paced rhythm was 0.06 mV (0.04–0.09 mV) [15]. Furthermore, in another study most of the targeted evoked delayed potentials (EDP) in a cohort of 15 patients undergoing VT ablation were localized within low voltage zones [16].

Our study offers an objective framework and a systematic quantitative method for utilizing the LV voltage map and LV segmentation to predict DEEP presence. The ability to use standard high-density voltage mapping to predict the location of DEEP+ sites offers a practical advantage in the procedure workflow. With careful interpretation of our results, the observations and methodology of our study could be carefully utilized at an early stage of the procedure, perhaps after the collection of voltage data and in combination with other parameters (like electrogram duration, conduction velocity, isochronal crowding, presence of late potentials, and wave frequency) to guide the operator into where to focus extra-stimulus functional mapping. As CT-based LV segmentation software are gaining popularity and is proposed for integration into some of the common mapping systems, these voltage metrics indeed can be provided by the mapping software. Hence the focus can be shifted into more detailed mapping of the areas identified. This might result in more efficient procedures and more feasible execution of S2 functional mapping, especially in challenging cases.

In fact, the need to utilize the voltage metrics to predict functional substrate is crucial, since the reliability of using the conventional voltage threshold alone to guide VT ablation is limited [17]. A study revealed that although VT channels (i.e. higher voltage channels within the scar) as identified by bipolar voltage were frequent (88%), they were not specific (30%) for locating the critical VT isthmuses. However, the localization of local abnormal potentials within a channel made it a much more specific predictor [18]. Furthermore, a study showed that low voltage areas detected by conventional voltage mapping still contain functionally viable tissue by Fluorodeoxyglucose Positron Emission Tomography (FDG PET). Ablation should be applied as sparingly as possible in these viable sites since they may not harbour functional substrate [19].

### Limitations

Despite the noted association between DEEP+ location and voltage metrics in our analysis, there are multiple limitations that must be considered. First of all, the small sample size may affect the power of the results. Secondly, the mapping density dispersion between cases might result in over- or underestimation of regional voltage metrics as well as the ability to detect DEEP points. Thirdly, the classification of DEEP status for each LV segment is inherently influenced by mapping density, regardless of the classification method/approach (e.g. number of EGM, a proportion-based, or surface area-based). The adopted method of classification based on an EGM-count threshold is simpler, more directly reproducible from the procedural data, technically feasible, and aligns with the clinical concept that the presence of multiple DEEP EGMs within a segment flags the surrounding region as a target for further investigation. Other classification methods such as surface-area (SA) based, would also be subject to technical limitations such as the fixed minimal diameter of the tag points representing a DEEP location, making accurate SA estimates infeasible. Fourthly, the analysis depends on co-registration between EAM and cardiac CT, which is subject to alignment deficiencies and might affect the number of mapping points displayed on each LV segment. Furthermore, the metrics calculation was based on LV segmentation into 17 segments of more or less similar size. The use of a greater number of LV segments with smaller sizes could have resulted in greater precision. However, the used 17 LV segments model is based on the widely validated 17-segment LV model based on the American Heart Association (AHA) consensus [20]. Finally, the AUCs of approximately 70% are modest in accuracy and suggest that these metrics alone may be insufficient for definitive diagnostic cutoffs, yet still meaningful to use in intraprocedural decision making (i.e. along other parameters). However, future prospective studies with a larger sample size and denser maps are warranted to further validate our findings with the applied methodology.

## Conclusion

Left ventricular regions harboring critical electrograms displaying a DEEP+ response tend to have lower regional voltage median and lower voltage IQR. These voltage metrics represent a component that, alongside other mapping parameters, might facilitate and predict the locations where further detailed functional mapping can be performed to identify the VT substrate.

## Data Availability

All data underpinning this study are available upon reasonable request to the corresponding author. To protect participant privacy, only anonymized data will be shared. Access will be granted for approved scientific inquiries.

## References

[1] Foth C, Gangwani MK, Ahmed I, Alvey H. Ventricular tachycardia. StatPearls. edn. Treasure Island (FL); 2025.

[2] Martin R, Hocini M, Haisaguerre M, Jais P, Sacher F. Ventricular tachycardia isthmus characteristics: insights from High-density mapping. Arrhythm Electrophysiol Rev. 2019;8(1):54–9. 10.15420/aer.2018.78.2 PMC6434507 30918668

[3] Nof E, Stevenson WG, John RM. Catheter ablation for ventricular arrhythmias. Arrhythm Electrophysiol Rev. 2013;2(1):45–52. 10.15420/aer.2013.2.1.45 PMC4711562 26835040

[4] Ciaccio EJ, Hsia HH, Saluja DS, Garan H, Coromilas J, Yarmohammadi H, Biviano AB, Peters NS. Ventricular tachycardia substrate mapping: what’s been done and what needs to be done. Heart Rhythm. 2025;22(9):2280–99. 10.1016/j.hrthm.2025.02.034 39988104

[5] Jackson N, Gizurarson S, Viswanathan K, King B, Masse S, Kusha M, Porta-Sanchez A, Jacob JR, Khan F, Das M, et al. Decrement evoked potential mapping: basis of a mechanistic strategy for ventricular tachycardia ablation. Circ Arrhythm Electrophysiol. 2015;8(6):1433–42. 10.1161/CIRCEP.115.003083 26480929

[6] Graham AJ, Orini M, Lambiase PD. Limitations and challenges in mapping ventricular tachycardia: new technologies and future directions. Arrhythm Electrophysiol Rev. 2017;6(3):118–24. 10.15420/aer.2017.20.1 PMC5610733 29018519

[7] Mills MT, Calvert P, Chiong J, Gupta D, Luther V. Dynamic voltage mapping of the Post-infarct ventricular tachycardia substrate: A practical technique to help differentiate Scar from borderzone tissue. Arrhythm Electrophysiol Rev. 2024;13:e16. 10.15420/aer.2024.26 PMC11539044 39507206

[8] Al-Sheikhli J, Winter J, Roca-Luque I, Lambiase PD, Orini M, Porta-Sanchez A, Dhanjal T. Optimization of decrementing evoked potential mapping for functional substrate identification in ischaemic ventricular tachycardia ablation. Europace. 2023;25(5). 10.1093/europace/euad092 PMC10228600 37032650

[9] Dodge Y. The concise encyclopedia of statistics. 1st ed. New York: Springer; 2008.

[10] Dekking M. A modern introduction to probability and statistics: Understanding why and how. London: Springer; 2005.

[11] Marchlinski FE, Callans DJ, Gottlieb CD, Zado E. Linear ablation lesions for control of unmappable ventricular tachycardia in patients with ischemic and nonischemic cardiomyopathy. Circulation. 2000;101(11):1288–96. 10.1161/01.cir.101.11.1288 10725289

[12] Misra S, Zahid S, Prakosa A, Saju N, Tandri H, Berger RD, Marine JE, Calkins H, Zipunnikov V, Trayanova N, et al. Field of view of mapping catheters quantified by electrogram associations with radius of myocardial Attenuation on contrast-enhanced cardiac computed tomography. Heart Rhythm. 2018;15(11):1617–25. 10.1016/j.hrthm.2018.05.031 PMC8262627 29870783

[13] Anter E. Limitations and pitfalls of substrate mapping for ventricular tachycardia. JACC Clin Electrophysiol. 2021;7(4):542–60. 10.1016/j.jacep.2021.02.007 33888275

[14] Tonko JB, Ruiperez-Campillo S, Cabero-Vidal G, Cabrera-Borrego E, Roney C, Jimenez-Jaimez J, Millet J, Castells F, Lambiase PD. Vector field heterogeneity as a novel omnipolar mapping metric for functional substrate characterization in scar-related ventricular tachycardias. Heart Rhythm. 2025;22(5):1218–28. 10.1016/j.hrthm.2024.10.066 39515493

[15] Martin R, Maury P, Bisceglia C, Wong T, Estner H, Meyer C, Dallet C, Martin CA, Shi R, Takigawa M, et al. Characteristics of Scar-Related ventricular tachycardia circuits using Ultra-High-Density mapping: A Multi-Center study. Circ Arrhythm Electrophysiol. 2018;11(10):e006569. 10.1161/CIRCEP.118.006569 30354406

[16] Sramko M, Abdel-Kafi S, van der Geest RJ, de Riva M, Glashan CA, Lamb HJ, Zeppenfeld K. New adjusted cutoffs for normal endocardial voltages in patients with Post-Infarct LV remodeling. JACC Clin Electrophysiol. 2019;5(10):1115–26. 10.1016/j.jacep.2019.07.007 31648735

[17] Hutchinson MD, Dhakal BP. Normal relativism: the impact of remodeling on electrogram amplitude after myocardial infarction. JACC Clin Electrophysiol. 2019;5(10):1127–9. 10.1016/j.jacep.2019.07.015 31648736

[18] Mountantonakis SE, Park RE, Frankel DS, Hutchinson MD, Dixit S, Cooper J, Callans D, Marchlinski FE, Gerstenfeld EP. Relationship between voltage map channels and the location of critical isthmus sites in patients with post-infarction cardiomyopathy and ventricular tachycardia. J Am Coll Cardiol. 2013;61(20):2088–95. 10.1016/j.jacc.2013.02.031 23524215

[19] Kettering K, Weig HJ, Reimold M, Schwegler AC, Busch M, Laszlo R, Gawaz M, Schreieck J. Catheter ablation of ventricular tachycardias in patients with ischemic cardiomyopathy: validation of voltage mapping criteria for substrate modification by myocardial viability assessment using FDG PET. Clin Res Cardiol. 2010;99(11):753–60. 10.1007/s00392-010-0182-2 20532538

[20] Cerqueira MD, Weissman NJ, Dilsizian V, Jacobs AK, Kaul S, Laskey WK, Pennell DJ, Rumberger JA, Ryan T, Verani MS, et al. Standardized myocardial segmentation and nomenclature for tomographic imaging of the heart. A statement for healthcare professionals from the cardiac imaging committee of the Council on clinical cardiology of the American heart association. Circulation. 2002;105(4):539–42. 10.1161/hc0402.102975 11815441

